# Autoindexing diffraction images with *iMosflm*

**DOI:** 10.1107/S0907444912048524

**Published:** 2013-06-13

**Authors:** Harold R. Powell, Owen Johnson, Andrew G. W. Leslie

**Affiliations:** a MRC Laboratory of Molecular Biology, Hills Road, Cambridge CB2 0QH, England

**Keywords:** autoindexing, multiple lattices, *Mosflm*, data processing

## Abstract

An overview of autoindexing diffraction images based on one-­dimensional fast Fourier transforms is presented. The implementation of the algorithm in the *Mosflm*/*iMosflm* program suite is described with a discussion of practical issues that may arise and ways of assessing the success or failure of the procedure. Recent developments allow indexing of images that show multiple lattices, and several examples demonstrate the success of this approach in real cases.

## Abstract 2

The principles of one-dimensional FFT-based autoindexing of diffraction images are described together with practical issues that may arise. A procedure for indexing multiple lattices as implemented in *iMosflm* is presented.

## 1. Introduction

Indexing is an essential first step in the processing of diffraction data from macromolecules using modern software suites. An alternative approach of integrating diffraction images first and then attempting indexing at a later stage has been implemented in the past (Kabsch, 1977 ▶), but proved to be significantly inferior owing to the difficulties in obtaining accurate estimates of the intensities of weak reflections. Indexing requires one or more diffraction images and a basic description of the experimental conditions (crystal-to-detector distance, radiation wavelength, detector orientation and the position of the direct beam). If two or more images are being used, the relative orientation(s) of the crystal is needed. The indexing provides a description of the crystal unit-cell parameters and the orientation of the crystal relative to a laboratory frame. On the basis of the shape of the derived unit cell, it may be possible to propose (as an initial hypothesis) the Laue symmetry of the crystal.

The advent of so-called ‘autoindexing’ procedures, in which the indexing can be carried out using one (or more) diffraction images without any prior knowledge of the unit-cell parameters or knowledge of the crystal orientation (*e.g* from its morphology), was a major advance in the development of automated or semi-automated data-processing software. Historically, the first autoindexing algorithms did require knowledge of (approximate) unit-cell parameters (Vriend & Rossmann, 1987 ▶; Kabsch, 1988 ▶), but these were rapidly superseded by improved procedures where this was not required (Kim, 1989 ▶; Higashi, 1990 ▶; Kabsch, 1993 ▶; Steller *et al.*, 1997 ▶; Sauter *et al.*, 2004 ▶). These improved algorithms form the basis of autoindexing in all modern software suites. However, two distinct approaches are employed. Both rely on a mapping of spot positions in the diffraction image to scattering vectors in reciprocal space (see §[Sec sec2]2). In the *XDS* program suite, difference vectors derived from these reciprocal-space vectors are used in indexing (Kabsch, 1993 ▶), while other widely used software suites use an approach based on a fast Fourier transform (FFT) of the reciprocal-space vectors either in three dimensions (Campbell, 1998 ▶; Otwinowski & Minor, 2001 ▶) or in one dimension (Steller *et al.*, 1997 ▶). The difference-vector approach used in *XDS* has been described in detail and will not be discussed here. Both difference-vector-based and FFT-based procedures are very powerful and there is no clear evidence to suggest that either method is intrinsically superior to the other. Enhancements to handle diffraction images from crystals with a pseudotranslation or to allow the robust identifi­cation of outliers have also been described (Sauter & Zwart, 2009 ▶; Sauter & Poon, 2010 ▶).

In this paper, we describe the general principles of one-dimensional FFT-based autoindexing, particularly with reference to its implementation in *Mosflm* (Powell, 1999 ▶) and *iMosflm* (Battye *et al.*, 2011 ▶). Ways to assess the success or failure of the indexing are described, along with a discussion of some of the practical issues that can influence this. Finally, the results of recent work aimed at indexing diffraction images that show multiple lattices are presented.

## 2. One-dimensional FFT-based indexing

**Figure 1:**
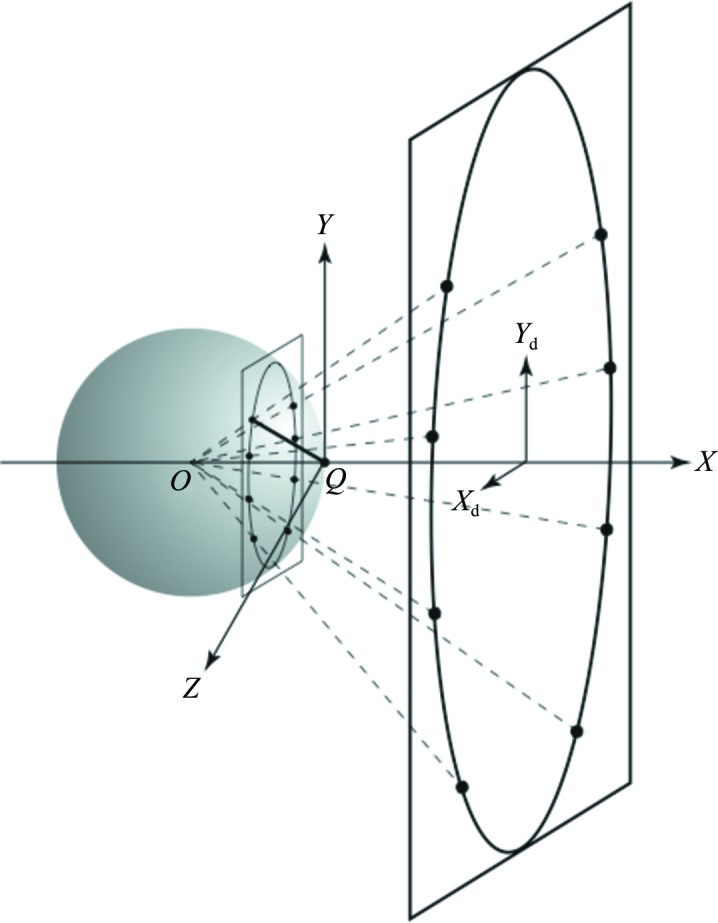
The Ewald sphere construction. The crystal is located at the centre of the Ewald sphere (*O*), the X-ray beam is along the *X* axis and the origin of the reciprocal lattice (*Q*) is at the point where the X-ray beam exits the Ewald sphere. The laboratory coordinate frame is defined by the orthogonal set of axes denoted *X*, *Y*, *Z* and the spindle axis is parallel to *Z*. A set of six reciprocal-lattice points lying in a plane parallel to the *YZ* plane are shown as lying on the surface of the Ewald sphere, giving rise to a set of diffracted beams (dotted lines) that result in a set of spots that lie on a circle on the planar detector. *X*_d_, *Y*_d_ define the detector coordinate frame. The scattering vector for one of the reciprocal-lattice points is shown as a bold line.

All indexing procedures rely on the fact that a diffraction image recorded using the oscillation method (Arndt & Wonacott, 1977 ▶) is a distorted projection of reciprocal space (the mechanism of precession cameras enables them to produce an undistorted projection, but as they rely on having an accurately oriented crystal they are now very rarely used). The geometry of the Ewald sphere construction can be employed to map back measured spot coordinates to the coordinates of the reciprocal-lattice point (rlp) that gave rise to that spot (Fig. 1 ▶).

This mapping is only strictly valid when the rlp lies exactly on the surface of the Ewald sphere. In practice, a variety of effects (crystal mosaicity, wavelength dispersion, beam divergence) lead to the rlp having a finite size, and thus a diffraction spot can be observed when the (centre of) the rlp lies significantly away from the Ewald sphere. These errors could in principle be minimized by determining the experimental ϕ centroid (where ϕ denotes the spindle rotation angle) from a succession of images with a very small oscillation angle (fine slicing), but in practice this is rarely performed (although with the advent of very fast readout detectors this is now entirely feasible).

The uncertainty in the true ϕ value for the spots results in errors in the calculated scattering vectors, with the size of the error dependent primarily on the mosaicity of the crystal and the oscillation angle of the image. This in turn affects the robustness of the indexing, particularly for highly mosaic crystals or moderate mosaicity combined with large unit-cell parameters. In both of these cases adjacent lunes will overlap in the diffraction image, leading to ambiguities in the indexing.

The one-dimensional FFT-based indexing algorithm has been described in detail elsewhere (Steller *et al.*, 1997 ▶; Powell, 1999 ▶), but the general principles involved are summarized here. Using the geometry of the Ewald sphere construction, spot coordinates (relative to the direct-beam position) are converted to dimensionless reciprocal-lattice scattering vectors (**s**) using
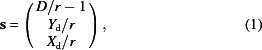


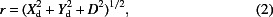
where *X*_d_, *Y*_d_ are the spot coordinates measured in the detector coordinate frame and *D* is the crystal-to-detector distance. The Ewald sphere radius is unity when working in dimensionless reciprocal-lattice units. As already stated, the true ϕ values for the spots are not known and so it is assumed that the ϕ values for all of the spots correspond to the midpoint of the oscillation range. If two or more images are being used, the scattering vectors must be referred to a common ϕ origin using
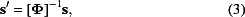
where the matrix [**Φ**] corresponds to a rotation around the spindle axis by an angle ϕ.

**Figure 2:**
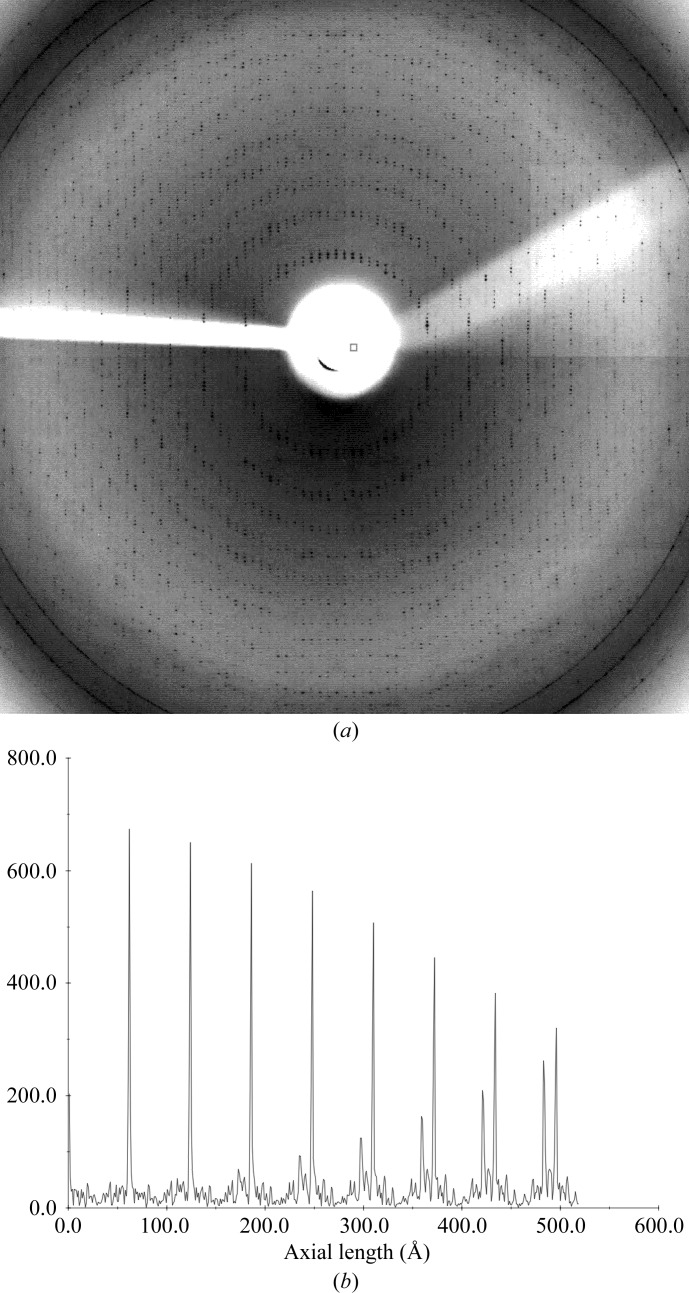
One-dimensional FFT indexing. (*a*) Diffraction from an aligned ribosome crystal. The crystal has been oriented so that a real-space axis lies along the X-ray beam, so the diffraction pattern shows spots that lie on a series of circular lunes centred on the direct-beam position. (*b*) An example of the Fourier transform of the scattering vectors projected along the direction of a real-space axis. In this example, the axial length is ∼62 Å.

To illustrate the underlying principles of one-dimensional FFT-based indexing, consider the diffraction pattern shown in Fig. 2 ▶(*a*), in which the crystal has been oriented so that one of the crystal axes is aligned along the X-ray beam direction (the *X* axis of the laboratory frame). The resulting diffraction pattern consists of a series of circular lunes centred on the direct-beam position. When the scattering vectors derived from the spot positions are projected onto the *X* axis, all of the projected scattering vectors corresponding to spots lying within the same circular lune will have the same length (within experimental error). The projected scattering vectors for all the spots will therefore lie in clusters at regular intervals along the *X* axis. A Fourier transform of these projected scattering vectors will give a series of large peaks owing to the periodicity of these clusters (Fig. 2 ▶ *b*). The Fourier transform also provides a mapping from reciprocal space (corresponding to the projected scattering vectors) to real space, and the first (non-origin) peak will occur at a spacing that provides the length of the crystal axis parallel to the X-ray beam.

Now consider the effect of projecting the scattering vectors onto an axis inclined at a small angle (a few degrees) to the *X* axis. In this case, spots lying in the same circular lune will give rise to projected scattering vectors of different lengths, so that the distribution of projected scattering vectors will tend towards a continuous distribution rather than regularly repeating clusters. In this case, the one-dimensional FFT of the projected scattering vectors will not yield any peaks of significant height because there is no underlying periodicity.

This result makes it possible to identify the directions of (low-order) real-space axes for a crystal in any arbitrary orientation, rather than the specific orientation described above. The one-dimensional FFT of the projected scattering vectors is carried out for a whole range of directions of the projection axis, sampling a complete hemisphere. Those directions that give rise to large peaks in the one-dimensional FFT are then known to correspond to the directions of real-space axes, and the position of the first non-origin peak gives the length of that real-space axis. In practice, after location of a significant FFT peak in the first coarse sampling of orientations, a grid search of the orientation with successively smaller step sizes is used to accurately determine the real-space axis orientation (Powell, 1999 ▶). This then gives a series of real-space vectors that are used in the next step. In *Mosflm*, by default, the 30 vectors with the largest FFT peaks are selected (if vectors are collinear, the longer one is eliminated). Three vectors from this list are chosen and used to derive the orientation matrix [**A**], which is in turn used to calculate the indices of all reflections using
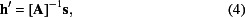
where the indices that comprise **h**′ will not, in general, be integral. The number of reflections for which any of the three derived indices (*h*, *k* or *l*) deviate from integers by less than a threshold (0.3) is determined. The process is repeated for all possible combinations of vectors, and the [**A**] matrix that yields the smallest number of rejections without having a significantly larger unit cell is chosen as the best solution.

**Figure 3:**
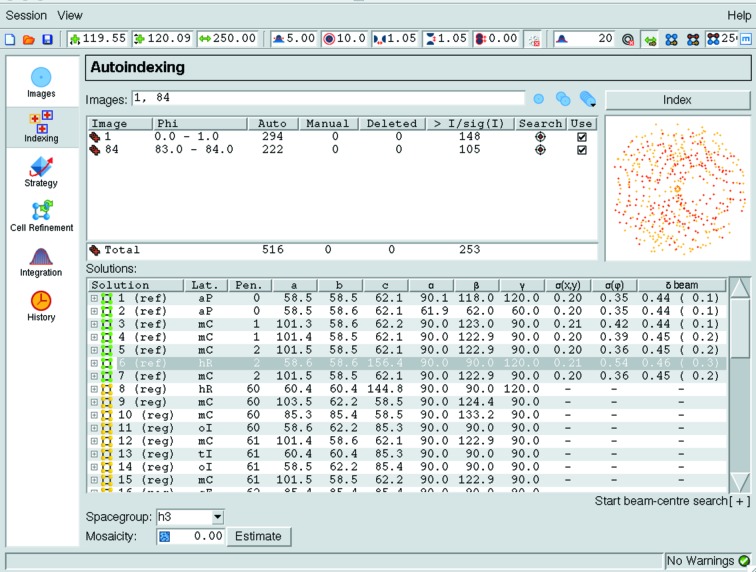
Indexing results as presented in *iMosflm*. For each solution the lattice type, distortion penalty and unit-cell parameters (in Å and degrees) are listed. For solutions with a penalty of less than 50, the r.m.s. errors in spot positions [denoted σ(*x*,*y*), in mm] and in ϕ [denoted σ(ϕ), in degrees] and the shift in the direct-beam position [denoted δ(beam), in mm] are given. The most likely solution is highlighted in grey.

Once the best solution has been determined, the reduced cell is calculated (Kim, 1989 ▶) and used to determine distortion penalties from higher symmetry lattices using the transformations for the 44 lattice characters tabulated in *International Tables for Crystallography* Volume *A* (see Appendix *A* [App appa]). The unit-cell parameters, direct-beam position and optionally the crystal-to-detector distance are refined against the observed spot positions for all lattices that have a distortion penalty of less than 50, applying the appropriate lattice constraints during the refinement, and the r.m.s. difference (r.m.s.d.) between the observed and the calculated spot positions is determined. Refinement of the crystal-to-detector distance is normally only appropriate for high-resolution data (>2 Å) because of the high correlation with the unit-cell parameters. The lattices are presented in a table (Fig. 3 ▶) and the program will highlight what is considered to be the most likely solution based on the distortion penalty and the r.m.s.d. value.

It is important to note that as the indexing is based entirely on spot positions, only information on the shape of the unit cell is obtained; the true symmetry can only be determined from the intensities (for example, by using the program *POINTLESS*; Evans, 2011 ▶) and reliable estimates of intensities are not available at this stage. Thus, it is easy to be misled by pseudo-symmetry: for example, a monoclinic cell with β ≃ 90° or an orthorhombic cell with two similar unit-cell edges. The r.m.s.d. value can be used to help identify pseudo-symmetry, and empirically it has been found that if a solution with a low penalty has an r.m.s.d. of >1.3 × r.m.s.d.^*P*1^ (where r.m.s.d.^*P*1^ is the r.m.s.d. value for the triclinic solution) then it is probably a pseudo-symmetric solution.

## 3. Requirements for success

The mapping from detector coordinates to reciprocal-lattice points relies on having accurate values for the wavelength, crystal-to-detector distance and, most importantly, the direct-beam position. In practice, the last of these is the most likely to be inaccurate. The indexing can be successful with only 30 spots, but a few hundred are ideal. The inclusion of even a relatively small number of ‘false’ spots arising from diffraction from ice, zingers or hot pixels can affect the indexing, so steps are taken to exclude possible false spots based on spot size, intensity and resolution, and normally only strong spots are used. The use of two or more images well separated in ϕ (90° is ideal) can significantly improve the success rate in difficult cases, and in all cases will result in better determined unit-cell parameters as a wider segment of reciprocal space is being sampled.

As mentioned earlier, large crystal mosaicity can also cause problems if this results in overlapping lunes. Incompletely resolved spots can cause issues with spot finding, leading to inaccurate spot positions. Although these errors are minimized by basing the spot finding on local maxima, it may be necessary to adjust the spot-finding parameters for challenging cases.

Finally, in cases where multiple lattices are present in the images the strongest lattice can often be indexed successfully by selecting only the strongest spots for indexing. Recent developments in indexing multiple lattices are described in §[Sec sec6]6.

## 4. Judging the success of indexing

Visual inspection is the best way to assess whether the indexing is correct. Assuming zero mosaic spread, not all spots will be predicted, but the pattern of lunes should match. Estimating the mosaic spread by pattern matching will usually make the comparison easier, but caution is required because if the prediction is wrong then the estimated mosaic spread is likely to be too large.

When visually inspecting the prediction, it is important to check that the chosen cell is not a sub-multiple of the true cell. For example, if pseudo-translational symmetry results in alternate strong and weak diffraction there is a risk that the corresponding unit-cell parameter will be half of its correct value if only the stronger spots are used in indexing.

The correct solution should have a low penalty (typically less than 20). The r.m.s.d. value is also a good indicator, although its actual value depends on a number of parameters. For well shaped diffraction spots, values of between 0.05 mm (low mosaicity and beam divergence and small detector pixel size, *e.g.* a CCD on a synchrotron beamline) and 0.2 mm (high beam divergence and/or large pixel size, *e.g.* an image plate on a laboratory source) are typical. However, for poor spot shape owing to a split crystal or very high mosaic spread the r.m.s.d. can be in excess of 1.0 mm for the correct solution, while for good spot shapes a residual this high would almost certainly represent an incorrect indexing. This serves to emphasize the importance of visual inspection.

## 5. Practical issues when indexing with *iMosflm*

Successful indexing depends on obtaining a reliable spot list, and it is therefore worthwhile inspecting the spots that are to be used. These are shown on the image display as red crosses for reflections to be used in indexing, while yellow crosses indicate spots that are below the current *I*/σ(*I*) threshold. The threshold is set automatically to 20 for images showing strong diffraction and is reduced to 10 or to 5 for weaker images. In cases where spots are poorly resolved or made up of multiple components (owing to a split crystal) it may be necessary to adjust the spot-finding parameters to obtain the best spot positions. The most useful parameter to adjust in the case of split spots is the minimum spot separation, which should be set to the approximate spot size (in millimetres).

### 5.1. Selection of images for indexing

By default, two images are used for indexing that are as close to a 90° separation in ϕ as possible. It is worth checking the quality of the second image, especially if this was collected at the end of a full data set, as radiation damage can result in very poor spot shape or in very weak diffraction, either of which could result in difficulties in indexing. In some cases crystal defects (*e.g.* disorder or multiple lattices) will only be visible in one of the two images, and selecting the better image alone can give successful indexing when using both fails. However, the use of two images is generally beneficial as it improves the success rate of indexing and will produce more accurate unit-cell parameters. For low-symmetry space groups (monoclinic or triclinic) indexing with a single image can give ambiguous results, with different unit-cell parameters giving an equally good prediction for that image; this ambiguity is often resolved if additional images are used. At present, no attempt is made to improve the estimate of the ϕ centroid when a diffraction spot is found at the same position on two images that abut in ϕ, and when multiple images are used for indexing it is recommended that they are chosen to be well separated in ϕ.

### 5.2. Direct-beam position

Inaccurate direct-beam coordinates are the most common cause of indexing failure when the images themselves are of good quality. The direct-beam position can be displayed on the image (as a green cross), making it trivial to check if it is in a sensible position, *i.e.* approximately centrally located within the shadow of the direct-beam stop. If necessary, the direct beam can be dragged to a reasonable starting position and a two-dimensional search carried out in which indexing is attempted over a grid of positions (by default ±2 steps of 0.5 mm from the starting position). A table summarizing the indexing results is presented, in which the correct solution is usually that which gives the lowest r.m.s.d. error. This search is generally more discriminating when two images (ideally 90° apart) are used. When selecting the correct solution it is necessary to reject those solutions with much larger unit-cell parameters than other solutions, as these can give low r.m.s.d. values even for an incorrect solution. If multiple starting direct-beam coordinates result in the same refined values this is a good indicator of success, but as in the standard indexing procedure visual inspection provides the best means of identifying the correct solution. When the unit cell is large, there can be multiple solutions with very similar r.m.s.d. values corresponding to a change in indexing of ±1 or even ±2 along a long axis. In these cases, assuming that the prediction looks good, it may be necessary to integrate a few degrees of data and then run the program *POINTLESS* (Evans, 2011 ▶) to determine the correct solution based on the values of the *R* factors or correlation coefficients.

### 5.3. Direction of spindle rotation

Another possible cause of indexing failure is when the direction of spindle rotation is opposite to that used on most beamlines. This is the case on some beamlines at MAX-­lab in Lund, the Advanced Photon Source at Argonne National Laboratory, the Shanghai Synchrotron Radiation Facility and the Australian Synchrotron in Melbourne. A useful indicator of this situation is that indexing using a single image is successful, but it fails when using two or more images. In addition, following successful indexing of a single image the prediction for the adjacent image (in ϕ) will not match. This is corrected by selecting the ‘Reverse direction of spindle rotation’ tick box in the Experimental Settings tab.

### 5.4. Images from crystals with a large mosaic spread

Diffraction images from crystals with a large mosaic spread (or from images with a large rotation angle) can present difficulties as this results in significant errors when mapping from spot positions to reciprocal-space vectors because of the assumption that the ϕ value for each spot is the mid-point of the oscillation range. In some cases, if a complete data set has been collected, there may be some images where the lunes are more clearly separated and indexing using these images may be successful. If only two reference images are available, it can be worthwhile collecting additional images at intermediate ϕ angles and possibly using a smaller oscillation angle. An alternative approach that is often successful is to set the *I*/σ(*I*) threshold to a very large value in the range 50–100, assuming that this still gives a reasonable number of useable spots. The basis for this is that the strongest spots will, on average, be those whose true ϕ values lie closest to the midpoint of the oscillation range of the image, and these will have the smallest errors in the assumed ϕ value.

### 5.5. When all else fails

In other cases where indexing fails, it is worth attempting indexing with a range of different values for the *I*/σ(*I*) threshold, both lower and higher than the default, or including additional images that are well separated in ϕ.

## 6. Indexing when multiple lattices are present

It is not uncommon for multiple distinct lattices to be present in diffraction images as the result of diffraction from different crystals with the same unit-cell parameters but in different orientations. The range of different orientations can vary considerably, with the most common case being small differences of 1–2° arising from split crystals, but larger differences of tens of degrees can also occur. The latter situation may arise when working with very small (∼10 µm or less) crystals and multiple crystals are present in the X-ray beam.

Approaches to indexing images with multiple lattices have previously been described in the literature. In the approach adopted in *LABELIT* (Sauter & Poon, 2010 ▶), it is assumed that an initial indexing succeeds with the full spot list, despite the presence of spots from more than one lattice. Outliers in the spot list are then identified based on the difference between their calculated and observed positions. A second indexing pass is then carried out based on spots that have been identified as outliers in the first pass. Essentially the same approach (but with a different algorithm for outlier identification) can be used in the *XDS* integration program (http://strucbio.biologie.uni-konstanz.de/xdswiki/index.php/Indexing). Within the *FABLE* software package (http://sourceforge.net/apps/trac/fable), indexing is based on the identification of reciprocal-lattice vectors based on prior knowledge of the unit-cell parameters and lattice type (Paithankar *et al.*, 2011 ▶). A similar approach has been adopted in software written by Anduleit and Stuart (D. I. Stuart, personal communication).

### 6.1. Multiple lattice indexing in *Mosflm*

An option to index multiple lattices has very recently been introduced into the *Mosflm*/*iMosflm* program suite. This implementation is similar to that adopted in *LABELIT* and also relies on successful indexing using the full spot list. While in principle this is an inherent weakness of this approach, in practice (see §[Sec sec6.3]6.3) the procedure has proved to be very successful. Increasing the number of vectors used for indexing from 30 might be worthwhile in difficult cases (and this is now possible from the *iMosflm* GUI), but was not required for any of the examples discussed in §[Sec sec6.3]6.3. Two criteria for the identification of outliers were investigated. The first was the difference between the observed and calculated spot positions (as used in *LABELIT*). The second was based on the indices assigned to spots using the orientation matrix obtained from the indexing. Because of errors in the reciprocal-space vectors derived from the spot positions (usually primarily owing to the uncertainty in the true ϕ coordinate), the calculated indices will not generally be exact integers. The deviation from integral values for the indices is used as a way of identifying outliers. In tests on a variety of images showing multiple lattices (see §[Sec sec6.3]6.3) the deviation from integral indices (Δ*hkl*) proved to be more successful than the deviation in spot positions in being able to index additional lattices, and this was adopted as the default criterion.

**Figure 4:**
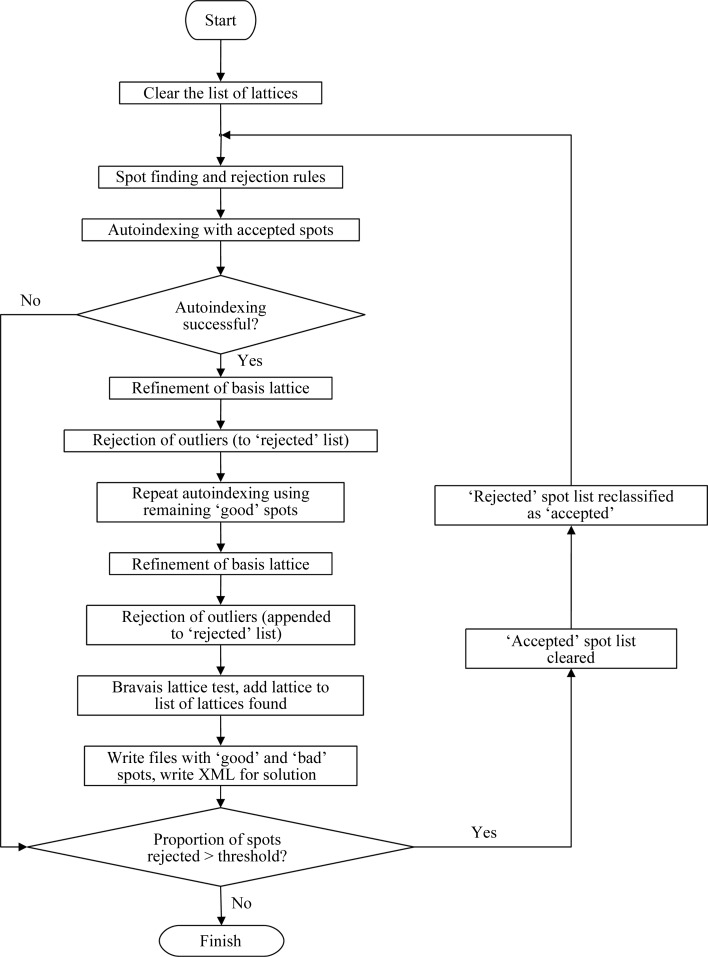
Flow diagram for indexing multiple lattices with *Mosflm*. See text for details.

*Mosflm* applies a maximum deviation Δ*hkl* of 0.3 from integral indices for conventional indexing (for a single lattice), but for the purposes of identifying multiple lattices a Δ*hkl* value of 0.2 gave a greatly improved performance (although this value can sometimes result in indexing failure in challenging single-lattice cases). Outliers based on this criterion are moved to a ‘rejected’ list, and a second indexing pass is carried out using the list of ‘accepted’ spots. Any additional outliers found are added to those in the rejected list. The rejected list is then used as input to a new indexing pass and the whole process is iterated until the rejected list contains less than a fixed percentage (10%) of the total number of spots found (Fig. 4 ▶).

### 6.2. Implementation

**Figure 5:**
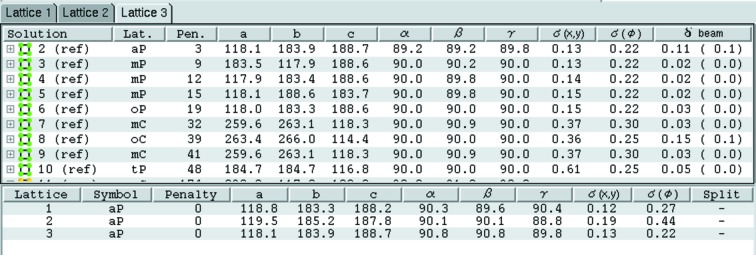
Indexing pane in *iMosflm* showing the results of multiple lattice indexing. The full sets of solutions for each detected lattice are displayed in the tabs headed Lattice 1, Lattice 2 *etc.* in the upper part of the pane. In the lower part the ‘best’ solutions for all detected lattices are shown. The final column will show the angular difference in orientation from the first lattice (not yet implemented). Selecting one of these solutions will result in the predicted reflections for that solution being displayed in the Image window.

Multiple lattice indexing has been introduced as an option on a pull-down menu in the Indexing pane of the *iMosflm* interface. The indexing is carried out as described above and the results are presented in the lower part of the pane with a separate tab for each lattice found (Fig. 5 ▶). Within this lower pane the lattice type, unit-cell parameters and positional residuals for the ‘best’ solution for each lattice found are listed, together with the difference in orientation from the first lattice. At this stage no attempt is made to enforce identical unit-cell parameters for the different lattices, although this could be imposed prior to the integration. Highlighting any of these solutions will result in the full list of possible solutions for that lattice being displayed in the upper part of the pane and will also update the predicted reflections displayed in the Image window. It is also possible to change the display of predicted reflections *via* the Lattice Selector in the Image display, making it very straightforward to check each of the different lattices found. A value of 0.2 for Δ*hkl* gave the best performance with the examples tested, but this parameter can be also be adjusted by the user.

### 6.3. Results

Examples of indexing several diffraction patterns showing multiple lattices are presented below. In some cases it was necessary to select two images that most clearly displayed the different lattices rather than the default procedure of using the first image and a second image separated by a 90° rotation in ϕ. All images were collected from cryocooled crystals at 100 K. In these examples, the correctness of the solutions was assessed based on their ability to predict most of the reflections present both on the images used for indexing and on images at other ϕ values. A full assessment would require successful integration of the different lattices and this is the aim of ongoing developments of the software.

#### 6.3.1. A triclinic unit cell, two lattices present

**Figure 6:**
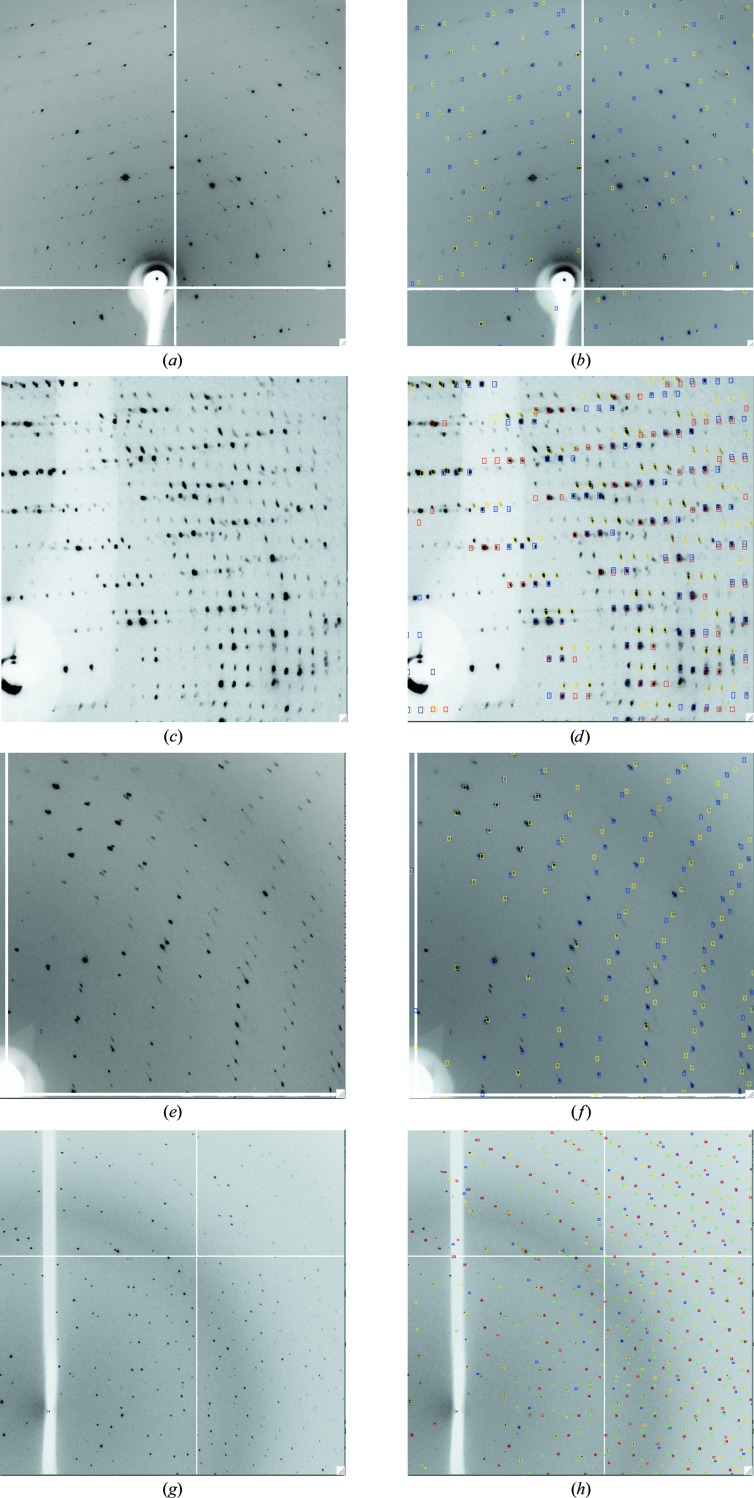
Examples of successful indexing of images that show multiple lattices. For each example, the original image is shown (*a*, *c*, *e*, *g*) and the same image with the predicted reflections (represented as square boxes) overlaid (*b*, *d*, *f*, *h*). The prediction boxes for different lattices are shown in different colours. No distinction is made between fully recorded, partially recorded or overlapped reflections or those with a ϕ width greater than 5° (the usual basis for colouring in the case of a single lattice). The mosaic spread has been set to 0.5° in all cases and no attempt has been made to optimize this. See text for further details.

Images were recorded using an ADSC Q4R CCD detector on beamline ID14-4 at the European Synchrotron Radiation Facility (ESRF) with an oscillation angle of 0.35°. Two triclinic lattices in widely differing orientations were identified using default parameter values and indexing with a single image either at ϕ = 0° or ϕ = 90° (Figs. 6 ▶ *a* and 6 ▶ *b*). In this example, only one lattice was found when indexing with both images together. A value of 0.25 for Δ*hkl* also gave two lattices (when using a single image), but only one lattice was found when Δ*hkl* was increased to 0.3.

#### 6.3.2. An orthorhombic unit cell with large unit-cell parameters

Diffraction data were collected using a Rayonix MX300 CCD detector on beamline I24 of the Diamond Light Source (DLS) from a crystal with orthorhombic symmetry and unit-cell parameters *a* = 118, *b* = 182, *c* = 188 Å. The oscillation angle was 0.5°. In this case, an image that shows the clearest separation of the different lattices was chosen for indexing and three different lattices were identified (Figs. 6 ▶ *c* and 6 ▶ *d*). The rotation angles relating the second and third lattice to the first were 1.8° and 3.5°, representing a ‘split-crystal’ scenario. Only two lattices were found when Δ*hkl* was increased to 0.25. Some spots were not predicted by any of the lattices found and therefore it seems possible that additional lattices are present.

#### 6.3.3. A monoclinic unit cell

Data were collected using an ADSC Q4R detector on beamline ID14-4 at the ESRF with an oscillation angle of 1.0°. Indexing with two images at ϕ = 0° and ϕ = 90° gave two lattices in very different orientations using all default parameter values (Figs. 6 ▶ *e* and 6 ▶ *f*). Indexing with single images was also successful. Increasing Δ*hkl* to 0.25 or 0.3 also resulted in two lattices when both images were used.

#### 6.3.4. A monoclinic unit cell and four lattices

This was the most challenging example because of the large number of lattices present. Images were collected on beamline I04 at DLS using an ADSC Q315R detector with an oscillation angle of 2.0°. It was essential to select images used for indexing that showed the clearest separation of the lattices, as spot overlap on some images gave poor results in the spot-finding step. It was not possible to index the lattices from a single image, but two images separated by 40° in ϕ led to successful indexing using default parameter values (Figs. 6 ▶ *g* and 6 ▶ *h*). The rotations relating the different lattices ranged from 0.8° to 9.4°. Increasing Δ*hkl* to 0.25 resulted in only two lattices being found.

### 6.4. Discussion

Despite the straightforward nature of the approach adopted, multiple lattice indexing has proved to be remarkably powerful for a range of different real-life examples. To improve the chances of success, it may in some cases be necessary to select images that show the distinct lattices most clearly on visual inspection, and the Δ*hkl* parameter may need to be adjusted within the range 0.2–0.3. Probably the greatest weakness of the current approach is that it relies on being able to perform the initial indexing step using the full spot list. However, in practice the one-dimensional FFTs of the scattering vectors show remarkably clear peaks indicating real-space lattice vectors even when four different lattices are present. In very challenging cases it may be possible to apply a filter to the projected scattering vectors, eliminating those that do not belong to obvious clusters, prior to calculating the one-dimensional FFT; this may further improve the success rate.

The ability to identify the different lattices depends significantly on their separation in orientation. Where this is only 1–2° (corresponding to the split-crystal scenario) there may well be difficulty in determining the true spot centroids for spots belonging to different lattices as they may be partially overlapped, especially at low resolution. This may explain why additional lattices could not be identified in the case described in §[Sec sec6.3.2]6.3.2. Further work is required to find the best approach in such cases.

Even when the multiple lattices have been correctly identified, the subsequent integration of the images taking proper account of all lattices present is challenging. Work is currently in progress on implementing the integration of multiple lattice data with *Mosflm*/*iMosflm*.

## 7. Program availability

The multiple lattice indexing version of the *Mosflm* suite is currently available from the authors as a beta release. The next major release of the program will include the multiple lattice indexing options and will also be available from the *CCP*4 website (http://www.ccp4.ac.uk).

## Appendix A Calculation of the distortion penalties

The distortion penalties are calculated as in the *DPS* program (Steller *et al.*, 1997 ▶), but for completeness this is described in detail below. The derivation derives from an analysis in terms of the 44 lattice characters (see Wolff, 2006 ▶).

For a reduced cell defined by the vectors **a**, **b**, **c**, penalties for the different lattice numbers presented in the autoindexing table of results are calculated based on a ‘goodness-of-fit’ parameter denoted Ω_tot_ that is made up of three components,

The first of these can be written as

where

Ω_gen2_ depends on the cell type, which in turn is defined by the value of *T*, where

For *T* > 0 (type I)

and for *T* ≤ 0 (type II)

Ω_group_ is calculated according to which of four groups the characteristic lattice belongs. These groups correspond to *A* = *B* = *C* (group 1), *A* = *B* (group 2), *B* = *C* (group 3) or no conditions (group 4), where

For group 1,

For group 2,

For group 3,

For group 4,

The final component depends on the lattice number and is given by

where *d* is the parameter for the corresponding lattice number under the column headed *D* in Table 9.2.5.1 of *International Tables for Crystallography* Volume *A*, *e* is the parameter under column *E* and *f* is the parameter under column *F*.

Values of Ω_tot_ for the different lattices are normalized to give a maximum value of 999.
